# Imaging Early Steps of Sindbis Virus Infection by Total Internal Reflection Fluorescence Microscopy

**DOI:** 10.1155/2011/535206

**Published:** 2011-11-24

**Authors:** Youling Gu, Yuanzheng Yang, Yuechueng Liu

**Affiliations:** Department of Pathology, University of Oklahoma Health Sciences Center, P.O. Box 26901, Oklahoma City, Ok 73190, USA

## Abstract

Sindbis virus (SINV) is an alphavirus that has a broad host range and has been widely used as a vector for recombinant gene transduction, DNA-based vaccine production, and oncolytic cancer therapy. The mechanism of SINV entry into host cells has yet to be fully understood. In this paper, we used single virus tracking under total internal reflection fluorescence microscopy (TIRFM) to investigate SINV attachment to cell surface. Biotinylated viral particles were labeled with quantum dots, which retained viral viability and infectivity. By time-lapse imaging, we showed that the SINV exhibited a heterogeneous dynamics on the surface of the host cells. Analysis of SINV motility demonstrated a two-step attachment reaction. Moreover, dual color TIRFM of GFP-Rab5 and SINV suggested that the virus was targeted to the early endosomes after endocytosis. These findings demonstrate the utility of quantum dot labeling in studying the early steps and behavior of SINV infection.

## 1. Introduction

Sindbis virus (SINV) is an alpha virus and a member of the Toga virus family that also includes Semliki Forest and Ross River viruses [1]. It was first isolated in 1952 from mosquitoes found in the Sindbis area in Egypt [2]. The decoded SINV genome revealed a single-strand RNA of 11.7 kb in size [3]. Upon cell entry, the viral RNA strand serves as a messenger RNA to translate four nonstructure genes that are essential for viral replication. In late stages of infection, structural genes are translated and synthesized from a subgenomic RNA. The resulting viral particles containing nucleocapsids of genomic RNA and capsid proteins, packaged with envelope proteins E1/E2, are formed and budded off as mature virus [4, 5]. The receptors that are involved in SINV entry into host cells remain poorly characterized. One of such receptors is the 67 kD high-affinity laminin receptor (LR), which has been shown to mediate SINV infection of BHK cells [6, 7]. In addition, it has been reported that heparan sulfate, a major cell surface component, is directly involved in SINV infection of cultured cells [6, 8]. Little is known regarding viral attachment and movement on host cell surfaces during early stages of SINV infection.

SINV has long been used as an experimental model for studying encephalitis because it causes encephalomyelitis in neonatal mice [9]. Moreover, since SINV has a broad host range and can deliver efficient gene expression, vectors based on the virus have been developed for vaccine production and gene therapy purposes [10, 11]. In cancer therapy, SINV has been tested as a potential oncolytic reagent. It has been shown that a SINV strain (Toto1101) derived from the wild-type SINV was effective targeting and killing tumor cells of ovarian, colon, prostate, and liver cancer patients, leading to tumor regression in animal models [12, 13].

In recent years, quantum-dot- (Qdot-) based fluorescence labeling has become increasingly employed for imaging cellular events including single-molecule tracking and live-cell imaging [14–16]. Qdots are semiconductor nanocrystals with a broad excitation and emission spectra. One advantage of Qdot is its resistance to photobleaching, allowing prolonged exposure to excitation light. In the current study, we labeled SINV with biotin and conjugated the virus with Qdot 605. Using single-virus tracking, we were able to dissect the behavior and dynamics of individual SINV during the initial stages of infection, which showed a two-step, receptor-mediated cell attachment process.

## 2. Materials and Methods

### 2.1. Cell Culture and SINV Preparation

BHK-21 cells were cultured in MEM supplemented with 10% FBS and antibiotics. The cells were maintained in a humidified 37°C incubator with 5% CO_2_. For the construction of SINV-GFP expressing the green fluorescent protein (GFP), GFP cDNA (from pS65T-C1, Clonetech) was first cloned into a shuttle vector pH2J1Y which was modified from pH3′2J1 (kindly provided by Dr. Guangpu Li, University of Oklahoma, Ok, USA). The plasmid pH2J1Y contained a linker with additional multiple cloning sites, Kpn I, Sal I, EcoR V, Hind III, and Nhe I, which was inserted between the Xba I and Bam H1 sites of pH3′2J1. The cDNA encoding the GFP was excised by Nhe I and Bam H1 and ligated into pH2J1Y at same sites. An Apa I-Xho I fragment of pH2J1Y harboring the GFP cDNA was subcloned into the SINV vector pToto/3′2J. To construct SINV-C′-YFP, in which the 62–106 amino acid sequence of the capsid protein was replaced with EYFP, an Sph I and an Mlu I sites flanking the replaced capsid sequence were introduced into pToto/3′2J by PCR mutagenesis. EYFP with Sph I and Mlu I cloning sites was amplified by PCR using pEYFP-C1 as a template. Recombinant SINV was produced by transfection of BHK21 cells using capped RNAs derived from sp6 RNA polymerase transcription of Xho I-linearized plasmid templates. Viral stocks were obtained by harvesting the culture supernatants 30 hr after infection and aliquots were stored at −80°C until use. Viral titers were determined by plaque assay on BHK21 monolayers using crystal violet staining.

### 2.2. Biotinylation of SINV

SINV-GFP was collected in serum free MEM diluted with saline (1 : 1) at ~4 × 10^8^ pfu/mL and was labeled with biotin at room temperature or on ice. Briefly, the virus was mixed with 10 mM stock NHS-PEG4-Biotin (29 Å spacer arm, Pierce) in PBS (pH 8.0) at a final concentration of 300 *μ*M for 30 min at room temperature. The reaction was terminated by adding glycine to 0.1 M and incubating for 15 min at room temperature. For biotinylation on ice, SINV was incubated with NHS-PEG4-Biotin as described above for 90 minutes on ice. The reaction was terminated by addition of glycine and further incubation at room temperature for 20 minutes. We did not notice any significant differences in the efficiency of biotinylation and the infectivity of the resulting biotinylated SINV produced under the two labeling conditions. For western blot analysis of the labeled SINV, aliquots of SINV were heated at 100°C for 3 min in sample buffer containing 1% SDS, 10 mM EDTA, 10 mM DTT, 15% glycerol, 20 mM Tris-HCl, pH 6.8, and 0.01% bromophenol blue. The SINV proteins were separated by SDS-PAGE and transferred onto nitrocellulose membrane. An alkaline phosphatase-conjugated streptavidin (1 : 5,000, Sigma) was used to visualize biotinylated SINV envelope proteins.

### 2.3. SINV Infection, Fluorescence Microscopy, and Image Analysis

Biotinylated SINV was used to infect BHK-21 cells at MOI (multiplicity of infection) of 5–20. The virus was incubated with the cells at 4°C for 1 hour. After washing with PBS to remove unbound virus, the cells were further incubated with streptavidin-conjugated Qdot 605 (Invitrogen) for 60 minutes and washed 3 times with cold PBS. The cells were fixed with 3.7% formaldhyde in PBS for 30 minutes at room temperature. Fluorescent images were acquired and digitized for analysis. For live cell experiments, the infection was performed on ice or at 4°C for 1 hour before shifting to a 35°C chamber for microscopy.

For TIRFM, we used an Olympus IX71 equipped with a 60X (n.a. = 1.45) TIRF objective heated to 37°C. Cells were cultured in a home-made glass bottom dish and maintained at 35°C in an environmental control chamber (Olympus, USA) supplemented with 5% CO_2_. TIRF illumination was achieved using a 20 mW 488 nm laser source for excitation and a long-pass dichroic beam splitter (500 nm). GFP/YFP was viewed with an emission filter of 525/20 nm, and Qdot 605 was viewed with an emission filter of 600/40 nm. Emission filters were controlled with a Lambda 10 (Sutter Instrument, Novato, CA, USA) high-speed filter wheel. Images were captured with a CCD camera (Quantix 57, Photometrics, Tucson, Ariz, USA) air cooled to −25°C. The camera was controlled by IPlab 3.9.4 (Scanalytics, Fairfax, Va, USA) and analyzed with the IPlab software. For expression of GFP-Rab5, BHK-21 cells were transfected using Lipofectamine 2000 (Invitrogen) and pGFP-Rab5 (Dr. Guangpu Li, University of Oklahoma Health Sciences Center). The cells expressing GFP-Rab5 were imaged under TIRFM 24–48 hours after transfection.

## 3. Results

### 3.1. Labeling SINV with Qdot 605

**Figure 1:**
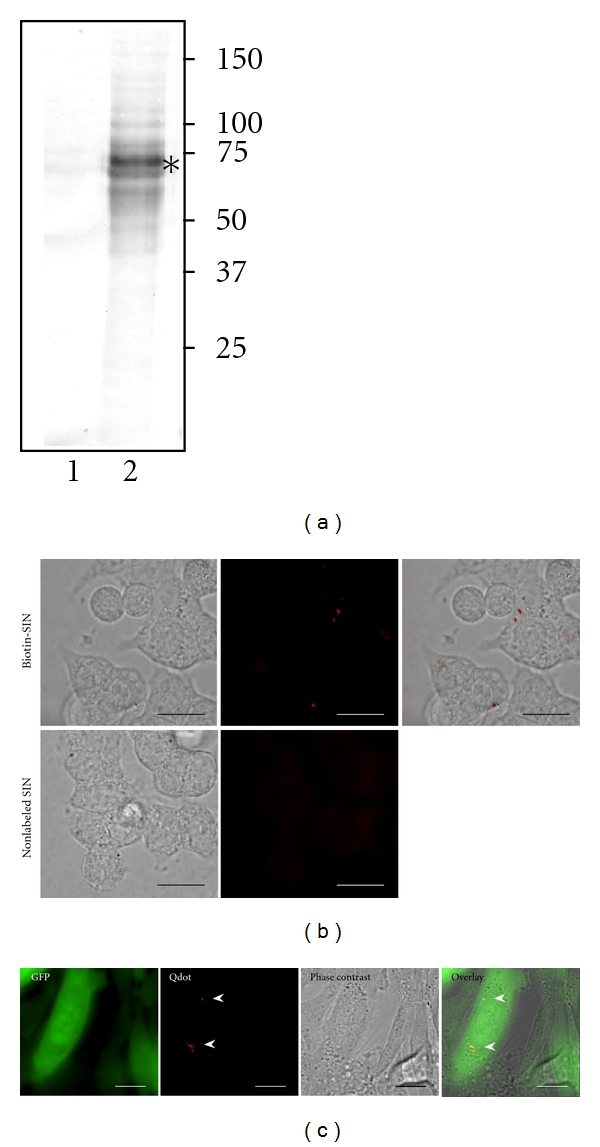
Biotinylation and Qdot labeling of SINV. SINV was labeled with NHS PEG4-biotin as described in Section 2. (a) Western blot analysis of biotinylated SINV using alkaline-phosphatase-conjugated streptavidin showing efficient labeling of the E1/E2 envelope proteins (indicated by *). Lanes 1: control unlabeled SINV and 2: biotinylated SINV. Molecular weight markers (kD) are labeled. (b) Biotinylated SINV labeled with Qdot 605 specifically bound target cells. Arrows indicate Qdot-labeled SINV. Bar = 25 *μ*m. (c) Biotinylated SINV labeled with Qdot 605 was active infecting target cells and expressing GFP. Images were taken 14 hours after infection. Arrowheads indicate Qdot signals. Bar = 25 *μ*m.

SINV was first conjugated to biotin linked through a 29 Å PEG arm. In order to achieve a near 100% labeling of SINV, an excessive amount of NHS-biotin at 300 *μ*M was used in the reaction. This was necessary for ensuring that the observed infection was indeed by the biotinylated SINV, not by the native and un-modified viruses. The conjugation reaction, however, reduced the infective titer of the virus from 4×10^8^ to 10^6^–10^7^ pfu/mL. Such a reduction was mostly due to the excessive chemical modification by NHS-biotin since viral titer was less affected when the concentration of NHS-biotin was reduced (data not shown). Western blot analysis showed that both the E1/E2 envelope proteins were labeled (Figure 1(a)). Moreover, the excessive crosslinking caused some E1/E2 proteins shifting to slightly higher apparent molecular weight (Figure 1(a)). When the biotin-SINV was preincubated with Qdot 605, a medium-sized quantum dot of 5–12 nm diameter, the virus-Qdot conjugate was still active infecting BHK-21 cells with a small reduction in infection activities (<2 fold). However, if we preincubated the cells with SINV for 1 hour at 4°C and then added Qdot, the reduction in infectivity became minimum. Specific binding to host cells by Qdot-labeled SINV was evident under fluorescence microscopy (Figure 1(b)). To determine if the Qdot-SINV was able to enter cells and carry out viral replication, we followed the expression of recombinant GFP encoded by the viral vector. BHK-21 cells were scored for GFP and Qdot 605 fluorescence 14 hours after infection, following the completion of first round of infection. Examination of randomly selected cells revealed that >70% cells expressing GFP (*n* = 500) were also positive for Qdot 605 (Figure 1(c)), which suggested that the Qdot-labeled SINV was able to enter the cells and continue to replicate and express viral genome.

### 3.2. Dynamics of Qdot-Labeled SINV on BHK Cell Surface

**Figure 2:**
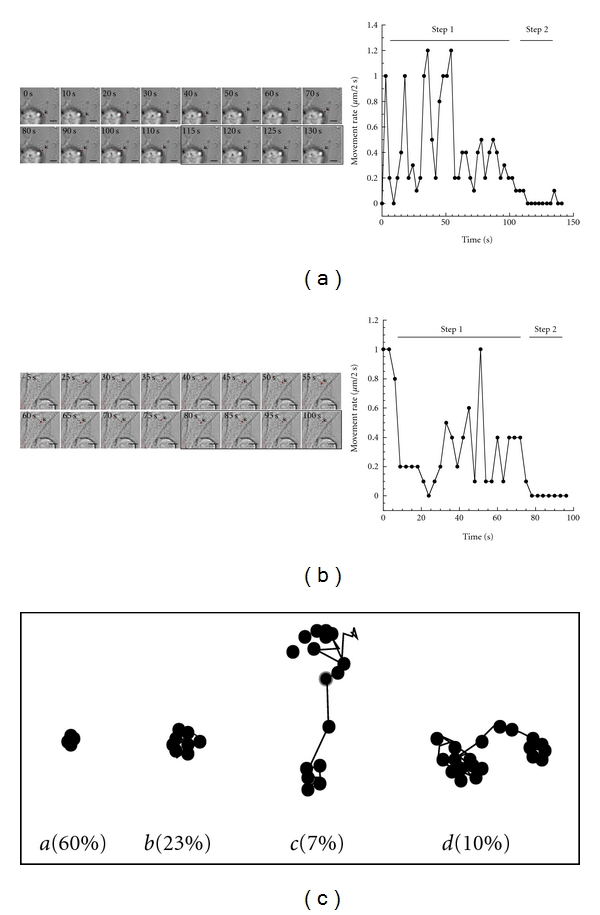
Single-particle tracking of a SINV virus binding to cell surface. Pseudocolor images showing that SINV attachment involved a two-step binding process. (a) A SINV-C′-YFP (arrow) was tracked under TIRF mode for 130 seconds. Its movement rate was measured manually frame-by-frame. Boxed images indicate no measurable movement by the virus. (b) Tracking of a SINV labeled with Qdot 605. (c) Four types of representative trajectories of Qdot-labeled SINV movement in BHK plasma membranes. *a*: immobile; *b*: mobile but confined in small areas (<0.5 *μ*m diameter); *c*: mobile through long distances (>1 *μ*m);* d*: mobile through medium distances (0.5–1 *μ*m). Scale bar = 10 *μ*m.

To study the dynamics of SINV on host cell plasma membranes, we used time-lapsed microscopy to record the movement of the virus in real time under TIRF. In order to synchronize viral attachment and to inhibit viral entry, we performed the incubation reactions at 4°C or on ice. This would presumably arrest SINV at surface attachment stage. The cell culture was then shifted to a 35°C environmental control chamber for microscopy. We first recorded the movement of the SINV-C′-YFP, which had a 44-amino-acid domain of the capsid protein replaced with EYFP. This virus was shown to retain the same infectivity as the wild-type virus generated from pToto/3′2J (data not shown). As shown in Figure 2(a), the virus moved rapidly in a random fashion at rates ranging from 0 to 600 nm/second. We were able to identify two types of virus-receptor association based on the rate of movement. One was brief and transient, which was exemplified by rapid movement of the virus. The other type of virus-receptor interaction appeared to be firm attachment, accompanied by the immobilization of the virus (Figure 2(a)). When we tracked SINV labeled with Qdot 605, the virus also exhibited the two types of cell association as the SINV-C′-YFP. The movement of attached Qdot-SINV appeared similar to that of SINV-C′-YFP, with a max rate of ~500 nm/second (Figure 2(b)). The SINV moved rapidly with a random trajectory. There were frequent associations and disassociations between the virus and cell surface. Most interactions appeared brief and transient. Full viral attachment became evident when the virus was firmly bound to the cell surface and became nearly immobile (Figure 2(b)). This finding suggested that Qdot did not significantly impact on the property of virus-receptor interactions. To investigate further into the behavior of Qdot-labeled SINV during cell surface attachment, we collected images from multiple cells (*n* = 200) and recorded 100 frames at 2 sec per frame rate for each cell. As illustrated in Figure 2(c), we observed 4 typical movements of SINV on cell surface. Nearly 60% of the viral particles were virtually immobile, indicating that they were likely bound to receptors nonspecifically or they might be functionally impaired from Qdot labeling. The other 40% of the SINV particles showed a heterogeneous lateral motility along the membranes. While some (23%) were confined in a small area (<0.25 *μ*m^2^), others (17%) were moving beyond several *μ*m in distance. The rate of SINV movement was also heterogeneous, ranging from 0 to 500 nm a second (Figure 2). It appeared that the virus often had preference for certain sites and would move in the vicinity. Moreover, movement would dramatically slow down once the virus settled on a particular site (Figure 2). These results suggested a two-step attachment process for the motile SINV. A first step involved a highly mobile receptor, and a second step involved the immobilization of the receptor/virus, perhaps prior to viral internalization (Figure 2).

### 3.3. Tracking SINV after Cell Entry

**Figure 3:**
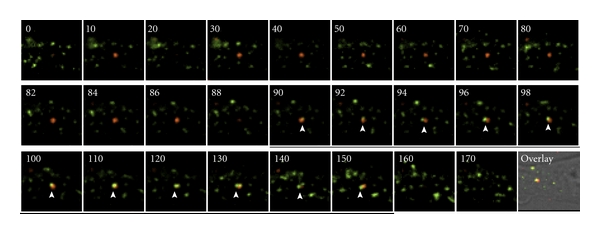
Dual color TIRFM of SINV and Rab-5 after endocytosis. A SINV virus after entering the cell was tracked (red). After approximately 90 seconds, it was associated with GFP-Rab5 (green). The association lasted ~60 second before the GFP-Rab5 dissociated and the SINV moved deeper into the cell. Time in seconds is labeled. Arrowheads indicate the association of Rab5. The last image shows an overlay composite picture of the cell.

SINV has been shown to undergo clathrin-dependent endocytosis after receptor binding [17]. However, the fate of the virus following endocytic reaction remains to be fully delineated. By tracking single SINV particles labeled with Qdot 605, we were able to determine that SINV was transported via Rab5-containing early endosomes. BHK cells expressing GFP-Rab5 were infected with Qdot-labeled SINV, and the virus was tracked at 35°C under TIRF. By taking the advantage of the superior optical sectioning capability of TIRFM, we were able to identify an internalized SINV based on its presence in the same optical plane with Rab5 under TIRF. As shown in Figure 3, SINV was targeted to Rab5-containing early endosomes after internalization. The co-localization of SINV with Rab5 lasted from 15 to 60 seconds, which indicated a rapid maturation of the early endosomes to late endosomes. Interestingly, about 40% of the SINV did not colocalize with GFP-Rab5 (data not shown).

## 4. Discussion

Quantum dots have been used in a wide range of applications including visualization of hepatitis C virus infection in human liver [18], tracking single SV40 virus [19], and imaging HER2 on tumor cells in real time [20]. Using Qdot-based single-virus tracking, we were able to begin dissecting the early events involving SINV infection. We have shown that, during a productive infection, SINV employed a two-step binding reaction to achieve high-affinity binding with its receptors. The first binding reaction resulted in a bound virus that was highly mobile and dynamic on the host cell surface. This binding often did not lead to viral internalization and could last from seconds to several minutes. The second step was high-affinity binding and attachment, which was accompanied by drastically reduced viral mobility. The immobilization of the virus might be a necessary step before viral endocytosis. After internalization, Qdot-SINV appeared to be transported via early endosomes containing Rab5 (Figure 3), which was consistent with previous reports on Semliki Forest virus [21]. However, approximately 40% of the Qdot fluorescence did not colocalize with Rab5-GFP (data not shown). One possibility for such a significant amount of Qdot-SINV without GFP-Rab5 co-localization was that they involved Rab5-negative endosome domains, similar to those reported for Semliki Forest virus [21]. Alternatively, due to Qdot's highly stable and strong fluorescence, it was difficult to record low levels of Rab5-GFP fluorescence, especially after long exposure (>2 minutes). Thus, the association of the Qdot-SINV with Rab5-negative compartment remains to be further investigated.

One advantage for quantum dots is that they are highly resistant to photobleaching. This allows prolonged excitation of the sample of interest, which in some instance requires minutes to hours of observation. In our experience using a 20 mW 488 nm laser, the Qdot 605 retained nearly 100% fluorescence intensity after 3 minutes of continuous illumination under TIR. In comparison, the EYFP showed a dramatic reduction of fluorescence emission after only 30 seconds of excitation and lost >90% of the intensity after 3 minutes. Consequently, tracking the SINV-C′-YFP was limited to <2 minutes, making it difficult to study the virus after internalization. With Qdot-labeled SINV, we were able to follow the virus for up to an hour, which greatly expanded data collection on viral infection process. Another benefit of quantum dots is that they have broad excitation spectra, which allows simultaneous excitation of multiple fluorophores. As shown in Figure 3, we were able to perform dual TIRF imaging using a single excitation source.

The limitation of quantum dots is their relatively large sizes, which range from 10 to 20 nm. When conjugated to biomolecules such as the E1/E2 envelope proteins of SINV, they could potentially interfere with the normal functions of the proteins. The greatest loss of viral infectivity was due to the initial biotinylation reaction. It appeared that some viruses were rendered inactive due to excessive biotinylation. Interestingly, binding of streptavidin-Qdot 605 had less adverse effect on SINV infection, which was further improved by adding the Qdot after SINV attachment to the cell surface. The relatively benign hindering effect upon Qdot binding to SINV suggested that, at an average 5–12 nm diameter, Qdot 605 did not significantly affect the infectivity of SINV. We believe this is likely due to the fact that Qdot 605 bound with SINV mostly at an 1 : 1 ratio. Nevertheless, conditions for the conjugation process can be further optimized to minimize hindering, which will make quantum-dot-based tracking a more widely used tool for investigating molecular details of viral infection.
